# Differential diagnosis and feature visualization for thyroid nodules using computer-aided ultrasonic diagnosis system: initial clinical assessment

**DOI:** 10.1186/s12880-022-00874-7

**Published:** 2022-08-30

**Authors:** Fang Xie, Yu-Kun Luo, Yu Lan, Xiao-Qi Tian, Ya-Qiong Zhu, Zhuang Jin, Ying Zhang, Ming-Bo Zhang, Qing Song, Yan Zhang

**Affiliations:** Department of Ultrasound, First Medical Center, Chinese PLA General Hospital, No. 28, Fuxing Road, Haidian District, Beijing, 100853 China

**Keywords:** Computer-aided diagnosis, Thyroid nodule, Ultrasonography, Diagnostic efficacy

## Abstract

**Background:**

To assess the diagnostic efficacy of the computer-aided ultrasonic diagnosis system (CAD system) in differentiating benign and malignant thyroid nodules.

**Methods:**

The images of 296 thyroid nodules were included in validation sets. The diagnostic efficacy of the CAD system was compared with that of junior physicians and senior physicians, as well as that of the combination diagnosis of the CAD system with junior physicians. The diagnostic efficacy of the CAD system for different sizes of thyroid nodules was compared.

**Results:**

The diagnostic sensitivity and accuracy of the CAD system were higher than those of junior physicians (83.4% vs. 72.2%, 73.0% vs. 69.6%), but the diagnostic specificity of the CAD system was lower than that of junior physicians (62.1% vs. 66.9%). The diagnostic accuracy of the CAD system was lower than that of senior physicians (73.0% vs. 83.8%). However, the combination diagnosis of the CAD system with junior physicians had higher accuracy (81.8%) and AUC (0.842) than those of either the CAD system or junior physicians alone, and comparable diagnostic performance with those of senior physicians. The Kappa was 0.635 in the combination diagnosis of the CAD system with junior physicians, showing good consistency with the pathological results. The accuracy (76.4%) of the CAD system was the highest for nodules of 1–2 cm.

**Conclusion:**

The CAD system can effectively assist physicians to identify malignant and benign thyroid nodules, reduce the overdiagnosis and overtreatment of thyroid nodules, avoid unnecessary invasive fine needle aspiration, and improve the diagnostic accuracy of junior physicians.

## Background

Thyroid nodule is one of the most common diseases at present, and its incidence has been rising yearly [1–3]. Along with the extensive application of high-resolution ultrasonography, the detection rate of thyroid nodules was up to 19–68% [4, 5], most of which are benign, and malignant ones only account for 7–15% [6, 7]. The National Comprehensive Cancer Network (NCCN) released the latest diagnostic and treatment guidelines for thyroid cancer in 2018, which highlighted the importance of differential diagnosis of benign and malignant thyroid nodules (http://guide.medlive.cn/.2018.5.22).

Both Chinese and international guidelines recommend ultrasonography as the preferred imaging examination method for detection and follow-up of thyroid nodules. Ultrasonography can not only identify malignant and benign thyroid nodules, but also assess preoperative and postoperative lymph node metastasis for malignant nodules [8–10]. However, due to the complexity and diversity of the ultrasound images of thyroid nodules, the ultrasound images of benign and malignant nodules have many similarities. Besides, subjective factors are still implicated in ultrasound diagnosis, and the differential diagnosis of some malignant and benign thyroid nodules is still difficult. Therefore, the ultrasound diagnostic accuracy for thyroid nodules varies greatly in different regions, at hospitals of different levels and among physicians with different experience. To reduce the variability in ultrasound-based diagnosis and improve the diagnostic accuracy, it is essential to develop a new accurate, convenient, efficient and noninvasive method to dispense with unnecessary fine-needle aspiration (FNA) biopsy and diagnostic surgery.

With the rapid development of big data and computer technology, Computer-aided Diagnosis (CAD) has achieved enormous progress in the field of medical imaging. By extracting massive features from imaging data to quantify diseases such as tumors, the CAD system can effectively solve the problem of quantitative assessment for benign and malignant tumors. At present, a variety of CAD software programs have been used in the clinical application including S-Detect, AI-SONIC and AmCAD-UT software [11–14]. AmCAD-UT, a Food and Drug Administration (FDA)-approved CAD software device, is utilized to not only differentiate benign and malignant nodules, but also diagnose the features of thyroid nodules with different thyroid imaging reporting and data system (TI-RADS) [15]. Previous studies have shown that AmCAD-UT system has comparable performance to physicians in terms of diagnostic efficiency [13, 15–17]. However, there are few no detailed reports focused on diagnostic efficacy of nodule size using AmCAD-UT system.

In this study, the AmCAD-UT system was used to analyze the image features of benign and malignant thyroid nodules and conduct the malignant risk assessment of thyroid nodules. Meanwhile the validity and reliability of the AmCAD-UT for assisting ultrasound diagnosis were evaluated.

## Materials and methods

### Subjects

From January to June 2018, 1000 patients received routine ultrasonography examination of thyroid nodules with defined surgical or fine needle aspiration pathological results in the PLA General Hospital. Then 400 cases were randomly selected according to the random number table method. 110 cases with Hashimoto's thyroiditis, Graves' disease, hypothyroidism, or poor quality images and unqualified images were excluded. Finally, a total of 290 patients with 296 nodules were included in the validation sets, with 158 nodules ≤ 1 cm, 106 nodules 1–2 cm, and 32 nodules ≥ 2 cm. Among them, 208 patients with 214 nodules underwent surgical resection, and pathology confirmed that 138 malignant nodules were identified as papillary thyroid carcinoma and 76 nodules were benign lesions. Of the 82 patients with 82 nodules who received FNA, 13 malignant nodules were classified by Bethesda as class VI, consistent with papillary thyroid carcinoma, and 69 benign nodules were classified by Bethesda as class II, consistent with benign lesions. Therefore, 151 malignant nodules and 145 benign nodules were included in this study.

### Methods

#### AmCAD-UT Detection System (CAD system)

The AmCAD-UT Detection System (CAD system) used in this study is a thyroid ultrasound image processing software (AmCad BioMed Corporation, Taiwan, China), which is compatible with Windows operating system. The physicians can select the region of interest (ROI) on the ultrasound images of thyroid nodules, and then the computer may process automatically the gray-scale images of the ROI, quantify image features, and achieve computer-aided ultrasound diagnosis.

#### Storage and recognition of ultrasound images

296 thyroid nodules images were obtained from 10 different ultrasonic instruments: iU-22 (Philips), iU Elite (Philips), ACUSON S2000 (Siemens), ACUSON SEQUOIA 512 (Siemens), Vivid E9 (GE), MyLab Twice (Esaote), HI VISION Ascendus (Hitachi), Supersonic (Supersonic Imagine), Resona 7 (Mindray), and Vinno 70 (VINNO). The above instruments are equipped with line array probes with a frequency of 4.5–13 MHz.

The original ultrasonic images of 296 thyroid nodules were all standard cross-sectional view images, and the depth and gray-scale gain of ultrasonic images and other parameters adjustment were all appropriate.

**Fig. 1:**
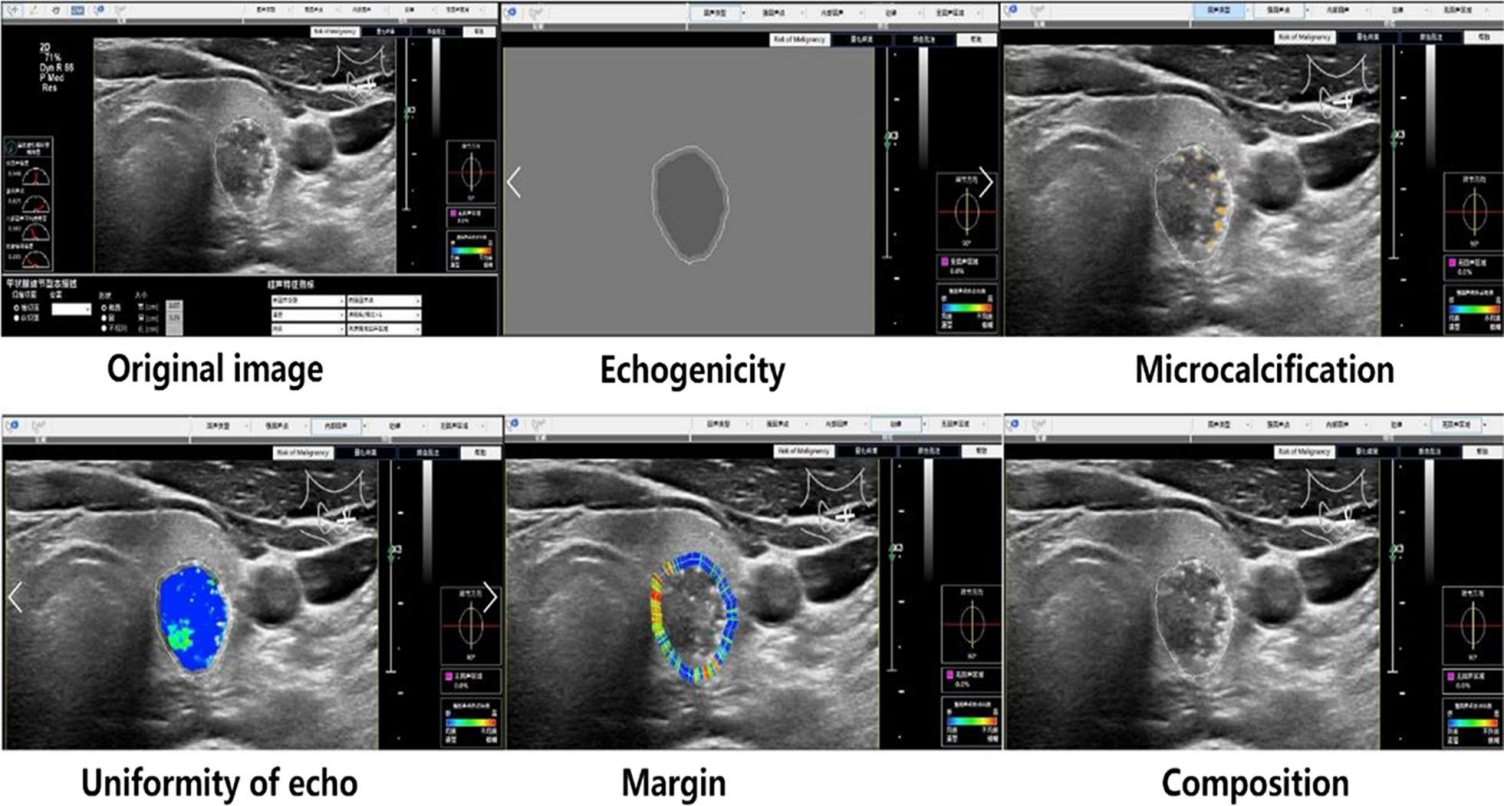
Automatic identification and quantization of ultrasound features of malignant thyroid nodules by the Am CAD-UT Detection System

**Fig. 2:**
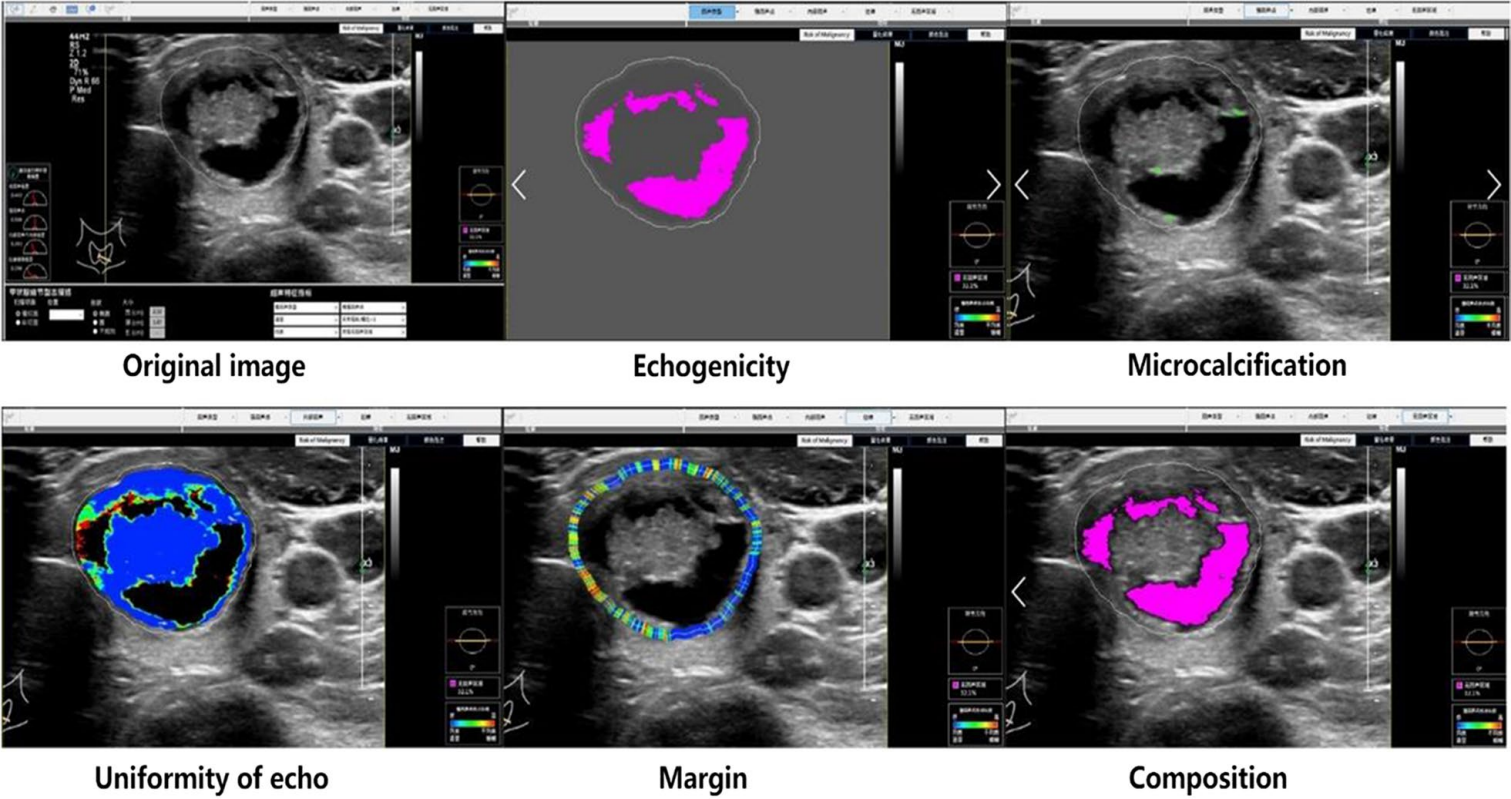
Automatic identification and quantization of ultrasound features of benign thyroid nodules by the AmCAD-UT Detection System

A physician more than 5-year experiences in ultrasound diagnosis marked the anteroposterior diameter and transverse diameter of the original standard images of 296 thyroid nodules with the cursor. Patient data and characteristics were blinded in AmCAD-UT application. Then the computer automatically traced the initial contour of ROI, identified the ultrasound image features of the thyroid nodules, then labeled and quantified the features in different colors, including echo type of the nodules, solid/cystic nature, uniformity of echo, nodular morphology, aspect ratio, and with or without microcalcification (Figs. 1, 2). Finally, the CAD system automatically generated the malignancy risk assessment report and suggestions of thyroid nodules based on 2015 ATA Guidelines [5], 2017 ACR TI-RADS Guidelines [8], 2016 AACE/ACE/AME Guidelines [18] and 2011 Kwak TI-RADS Guidelines [19] in the program.

#### Comparison of the diagnostic efficacy

By applying 2017 ACR TI-RADS Guidelines [8], 296 thyroid nodules were individually diagnosed by junior physicians engaged in ultrasound diagnosis for less than 5 years, and senior physicians engaged in ultrasound diagnosis for more than 10 years physicians. Both junior and senior physicians were blinded to all clinical, pathological, and CAD results. After analysis of the images independently, junior physicians gave the revised joint benign and malignant diagnosis recommendations of the nodules referring to the detailed diagnosis of the nodule characteristics by the CAD system in the combination of CAD system with junior physicians group.

### Statistics

SPSS 24.0 software (IBM Corporation, USA) was used, and *P* < 0.05 was considered statistically significant difference. The pathological results were taken as the gold standard. The diagnostic efficacy was assessed for the four diagnostic methods based on accuracy, sensitivity, specificity, positive predictive value and negative predictive value and AUC. The diagnostic efficacy of the CAD system for different sizes of thyroid nodules was also estimated. Kappa value was used to evaluate the consistency of pathological results and each diagnostic method.

## Results

The general information of 296 thyroid nodules in the validation sets was shown in Table 1. There were 151 malignant nodules and 145 benign nodules.

**Table 1:** General information of 296 thyroid nodules

	Pathology (n = 296)	
	Malignant: 151 (51%)	Benign: 145 (49%)
Gender		
Male	23 (7.8%)	56 (18.9%)
Female	128 (43.2%)	89 (30.1%)
Age		
< 55	119 (40.2%)	89 (30.1%)
≥ 55	32 (10.8%)	56 (18.9%)
Nodule size		
≤ 1 cm	82 (27.7%)	76 (25.7%)
1–2 cm	56 (18.9%)	50 (16.9%)
≥ 2 cm	13 (4.4%)	19 (6.4%)

Four different methods, CAD system, junior physicians, senior physicians, and combination of CAD system with junior physicians, were used to carry out the diagnosis of 296 thyroid nodules (Table 2). The diagnostic sensitivity, positive predictive value, negative predictive value, accuracy and AUC of the CAD system were all higher than those of junior physicians (83.4% vs. 72.2%, 69.6% vs. 69.4%, 78.3% vs. 69.8%, 73.0% vs. 69.6%, 0.728 vs. 0.695, respectively). Only the specificity of the CAD system was lower than that of junior physicians (62.1% vs. 66.9%).

**Table 2:** Comparison of diagnostic efficacy of four different methods on 296 thyroid nodules

Group	Sensitivity%	Specificity%	Positive predictive value%	Negative predictive value%	Accuracy %	AUC (95% CI)
CAD system	83.4	62.1	69.6	78.3	73.0	0.728 (0.673–0.777)
Junior physicians	72.2	66.9	69.4	69.8	69.6	0.695 (0.640–0.747)
Senior physicians	85.4	82.1	83.2	84.4	83.8	0.858 (0.808–0.909)
CAD system combined with junior physicians	84.1	79.3	80.9	82.7	81.8	0.842 (0.790–0.894)

The diagnostic sensitivity, specificity, positive predictive value, negative predictive value, accuracy and AUC of the CAD system were all lower than those of senior physicians (83.4% vs. 85.4%, 62.1% vs. 82.1%, 69.6% vs. 83.2%, 78.3% vs. 84.4%, 73.0% vs.83.8%, 0.728 vs. 0.858, respectively).

However, it was gratifying that the sensitivity (84.1%), specificity (79.3%), positive predictive value (80.9%), negative predictive value (82.7%), accuracy (81.8%) and AUC (0.842) of the combination of CAD system and junior physicians were all higher than those of either alone, and were close to the diagnostic efficacy of senior physicians.

The consistency of the diagnostic results from each of the four methods with the pathological results was tested (Table 3). The Kappa values of the CAD system, junior physicians and senior physicians were 0.457, 0.391 and 0.675, indicating moderate, low and strong consistency with the pathological results, respectively. Interestingly, the Kappa value of the combination of CAD system with junior physicians was 0.635, showing a stronger consistency with the pathological results than that of junior physicians alone.

**Table 3:** Consistency between four diagnostic methods and pathological results

	CAD system versus pathology	Junior physicians versus pathology	Senior physicians versus pathology	CAD system combined with junior physicians versus pathology
Kappa	0.457	0.391	0.675	0.635
*p*	0.000	0.000	0.000	0.000

In addition, the comparison of the diagnostic efficacy of the CAD system for different sizes of thyroid nodules was shown in Table 4. The accuracy (76.4%) and AUC (0.756, 95%CI: 0.663–0.835) of the CAD system was the highest for nodules of 1–2 cm. By contrast, the accuracy (70.9%) and AUC (0.706, 95% CI 0.628–0.776) of the CAD system was the poorest for nodules ≤ 1 cm, and the diagnosis accuracy (71.9%) and AUC (0.751, 95%CI: 0.567–0.886) was in the middle for nodules ≥ 2 cm.

**Table 4:** Comparison of diagnostic efficacy of CAD system on thyroid nodules of different sizes

Group	Sensitivity%	Specificity %	Positive predictive value%	Negative predictive value%	Accuracy %	AUC (95% CI)
≤ 1 cm	78.0	63.1	69.6	72.7	70.9	0.706 (0.628–0.776)
1–2 cm	89.2	62	72.4	83.8	76.4	0.756 (0.663–0.835)
≥ 2 cm	92.3	57.9	60.0	91.7	71.9	0.751 (0.567–0.886)

## Discussion

Differential diagnosis of benign and malignant thyroid nodules is of high clinical importance [20]. However, because of the subjective nature of the ultrasonic diagnosis and the similar imaging features of benign and malignant thyroid nodules, the ultrasonic diagnostic accuracy at different levels of hospitals and physicians varies greatly. Based on the current status of ultrasound diagnosis for thyroid nodules, along with the rapid development of computer technology and the intersection of multidisciplinary fields, the integration of ultrasound diagnosis and computer technology has been an inevitable trend.

Computer-aided diagnosis system can accomplish tumor segmentation, feature extraction and model establishment by extracting a large amount of image feature information from ultrasound images. CAD system can assist physicians to make more accurate and faster diagnosis by deeper mining and analysis of massive image data. Therefore, CAD system has gradually become a new diagnosis pattern as it can improve the diagnostic accuracy and work efficiency for physicians.

In this study, AmCAD-UT Detection System (CAD System) was evaluated. The results showed that the CAD system had the diagnostic sensitivity of 83.4%, specificity of 62.1%, positive predictive value of 69.6%, negative predictive value of 78.3%, accuracy of 73.0% and AUC of 0.728. It was found that the detection ability of the CAD system for malignant nodules was higher than that of junior physicians; and the negative predictive value of the CAD system was higher than that of junior physicians. As a method for screening diseases, the CAD system is very effective and appropriate. Moreover, when the CAD system was combined with junior physicians, the diagnostic sensitivity (84.1%), specificity (79.3%), positive predictive value (80.9%), negative predictive value (82.7%), accuracy (81.8%) and AUC (0.842) were all higher than those of either alone. This combination diagnostic method can compensate for the lower specificity (62.1%) of the CAD system alone and also for the lower sensitivity (72.2%) of junior physicians alone. Besides, the consistency between the combination diagnosis and the pathological results were strong (Kappa value 0.635), and the diagnostic accuracy of the CAD system combined with junior physicians was close to that of senior physicians (81.8% vs. 83.8%). Furthermore, the CAD system has the optimal diagnostic efficacy for nodules of 1–2 cm.

From previous studies on the computer-aided ultrasound diagnosis system of thyroid nodules [21, 22], most studies were only focused on the validation of the diagnostic efficacy of the CAD system [11, 23, 24]. In this study, we not only compared the performance of the CAD system with that of different levels of physicians, but also assessed the diagnostic efficacy of the combination of the CAD system with junior physicians. Besides, in order to verify the diagnostic efficacy of the CAD system in different sizes of thyroid nodules, we also categorized 296 nodules into three groups by size, and our results also showed that the accuracy and AUC of the CAD system was the highest for nodules of 1–2 cm, and the reproducibility was good. The above is the highlight of this study, which better illustrates the application value of the CAD system in assisting physicians in ultrasonic diagnosis.

Nevertheless, the CAD system still has some challenges in processing and analyzing image data [25]. However, it is undeniable that the computer-aided diagnosis system is refactoring a new medical diagnosis model. Ultrasound images containing a lot of objective information and some of medically significant information, is hard to identify by naked eyes. Based on big data mining technology, the CAD system can explore more valuable image information in digital signal form and visualize, which can not only help doctors in primary hospitals to obtain more accurate diagnosis, improve the diagnosis efficiency of doctors, but also contribute to the teaching practice of ultrasound imaging medicine.

There were several limitations to this study. First, only patients with papillary thyroid carcinoma were included in the current study. Second, CAD system was used to assess the thyroid nodule’s malignancy risk only based on trasverse image. In order to further verify the diagnostic efficacy of CAD system in differentiating thyroid nodules, other malignant tumors including follicular and medullary thyroid carcinoma will be included, and then the at least two images of significant longitudinal and trasverse scanning was used for analysis in the future.

## Conclusions

In conclusion, with the help of the CAD system, the variability of diagnosis could be reduced among physicians of different experience levels, meanwhile, the diagnostic accuracy of junior physicians could be improved. More importantly, with the ability of quick and autonomic learning, the CAD system can effectively compensate for the shortage of medical resources in some areas, especially in community hospitals and remote hospitals, and provide more reliable diagnostic services for patients.

## Data Availability

All data generated or analyzed during this study are included in this published article.
