# Correction: Differential diagnosis and feature visualization for thyroid nodules using computer-aided ultrasonic diagnosis system: initial clinical assessment

**DOI:** 10.1186/s12880-023-00970-2

**Published:** 2023-02-02

**Authors:** Fang Xie, Yu-Kun Luo, Yu Lan, Xiao-Qi Tian, Ya-Qiong Zhu, Zhuang Jin, Ying Zhang, Ming-Bo Zhang, Qing Song, Yan Zhang

**Affiliations:** Department of Ultrasound, First Medical Center, Chinese PLA General Hospital, No. 28, Fuxing Road, Haidian District, Beijing, 100853 China

**Correction to: BMC Medical Imaging (2022) 22:153** **https://doi.org/10.1186/s12880-022-00874-7**

Following the publication of the original article [1], we were informed that the funding information reported in the declarations was incorrect.

The statement "This study was supported by the National Natural Science Foundation of China [81771834] and the Natural Science Foundation of Beijing [Z181100001718017]" should be replaced by "None".

The original article has been revised as above.
